# Microcontroller Implementation of Support Vector Machine for Detecting Blood Glucose Levels Using Breath Volatile Organic Compounds

**DOI:** 10.3390/s19102283

**Published:** 2019-05-17

**Authors:** Matthew Boubin, Sudhir Shrestha

**Affiliations:** 1 Intelligent Systems Laboratory, Department of Engineering Science, Sonoma State University, Rohnert Park, CA 94928, USA; boubinmj@miamioh.edu; 2 Department of Electrical and Computer Engineering, Miami University, Oxford, OH 45056, USA

**Keywords:** breath disease detection, breath volatile organic compounds, diabetes, support vector machine, microcontroller implementation of SVM

## Abstract

This paper presents an embedded system-based solution for sensor arrays to estimate blood glucose levels from volatile organic compounds (VOCs) in a patient’s breath. Support vector machine (SVM) was trained on a general-purpose computer using an existing SVM library. A training model, optimized to achieve the most accurate results, was implemented in a microcontroller with an ATMega microprocessor. Training and testing was conducted using artificial breath that mimics known VOC footprints of high and low blood glucose levels. The embedded solution was able to correctly categorize the corresponding glucose levels of the artificial breath samples with 97.1% accuracy. The presented results make a significant contribution toward the development of a portable device for detecting blood glucose levels from a patient’s breath.

## 1. Introduction

In the United States, obesity, diabetes, cardiovascular diseases, and other metabolic disorders have been increasing in prevalence and severity at an extreme rate since the 1990s. In 2000, it was reported that less than 5% of US citizens were recorded to have been diabetic [1]. In 2016, the percentage of diabetic Americans rose 9.3% [2]. It is projected that, by 2030, 15.3% of Americans will have diabetes and that treatment of diabetes will cost the US economy a total of over 600 Billion US dollars per year [3]. With the prevalence of diabetes increasing at such an alarming rate, many new technologies are emerging to better monitor and manage blood glucose level. Among non-invasive glucose monitoring technologies, microwave sensors that are inspired by metamaterials, metasurfaces, nanoparticles, and graphene have shown great potentials in measuring glucose in aqueous solutions with a high sensitivity [4,5,6,7]. Recent research has focused on technologies such as meal detection [8], analysis of glucose in sweat [9], artificial pancreas technologies [10], and correlation between blood glucose levels and compounds present in breath [11,12]. Among these, researchers used variations of electronic-nose technologies to quantify glucose and other compounds. The evolution of electronic-nose application has been a recent trend in biomedical engineering and medical device industry. Several examples of electronic-nose applications include respiratory illness diagnosis [13], detection of cancers [14,15], detection of fungal diseases in harvested blueberries [16], and the general detection of volatile organic compounds (VOCs) in human breath [12]. With the availability of highly sensitive and low-cost VOC sensors, it has become affordable to create sensor-arrays that are able to detect VOCs in breath in parts-per-million (ppm) and even parts-per-billion (ppb) [17]. Refs [18,19] demonstrated the ability of SVM to differentiate between various types of tissues in a medical analysis. Specifically, SVM was trained to detect the difference between regular ovarian tissues and tissues containing ovarian cancer. These developments in sensing technology have made it possible to sense changes in VOCs in breath as changes in blood glucose levels of a diabetes patient.

This study intended to measure artificial breath samples that represent the breath of patients with high and normal blood glucose (BG) levels, and to correctly classify the breath samples represented by each level. Compared to non-invasive methods that measure glucose levels from an aqueous solution, the method presented in this paper measures VOCs in the breath and correlates it with the BG levels. SVM was trained to classify breath samples based on various features from a sensor array consisting of commercially available chemical sensors. The SVM training model was then compiled into a program run on an ATMega microprocessor to classify a breath sample in real time. The microprocessor implementation of the classification can be used to develop a portable biomedical device that can measure blood glucose levels from a patient’s breath sample instead of using a general purpose computer.

## 2. Design of Experiments

To train and test SVM, two sets of artificial breath samples, each with a unique VOC footprint that represented blood glucose range in a patient with diabetes, were created. One set represented a low BG levels (50–100 mg/dL) and the second set represented a high BG levels (180–240 mg/dL). Published data on diabetes breath analysis were reviewed for VOCs linked to blood glucose levels and their correlated concentrations [20,21,22]. Table 1 shows the breath VOCs that are consistently reported across publications as biomarkers of diabetes. Corresponding VOC concentrations and associated blood glucose levels are also shown. Among these VOCs, we chose acetone and ethanol as the two VOCs for further testing and analysis. This decision was informed by the extensive published work with acetone alone or acetone and ethanol for detecting diabetes from breath. For example, silicon tungsten oxide acetone sensors to estimate BG level is presented in [23]. A platinum functionalized tungsten oxide sensor for sensing acetone in exhaled breath is presented in [24]. Nanotubes and nanoparticles-based sensors for detecting acetone from breath are reported in [25,26]. A polymer-based sensor for detecting acetone and ethanol is reported in [27].

A test system was designed to ensure tests were conducted in a controlled and repeatable manner. The test system contained three 236 mL glass chambers connected to one another with chemical resistant tubing. A tank of ultra-clean air (20% oxygen and 80% nitrogen) was connected to a secondary flow control valve that permitted the air to flow at 0–1.5 L/min. As an effort to introduce carbon dioxide would increase the chamber setup complexity and also because CO${}_{2}$ does not have an impact on the sensors that are used in the presented study, air without CO${}_{2}$ was used. The receiving end of the flow control valve was connected to the first chamber, which contained water in which the clean air was percolated to simulate the humidity of a breath sample. Each compound or combination of compounds simulating a BG level was introduced into the second chamber. The sensor array was placed in the third chamber. The sensor array consisted of four commercially obtained VOC sensors. However, the responses from two of the sensors were minimal to the concentration levels used in this study and those responses, when included, did not provide any significant improvement in the SVM training. Thus, data from only two sensors, termed as Sensor-1 and Sensor-2, were utilized and presented in this paper.

**Figure 1:**
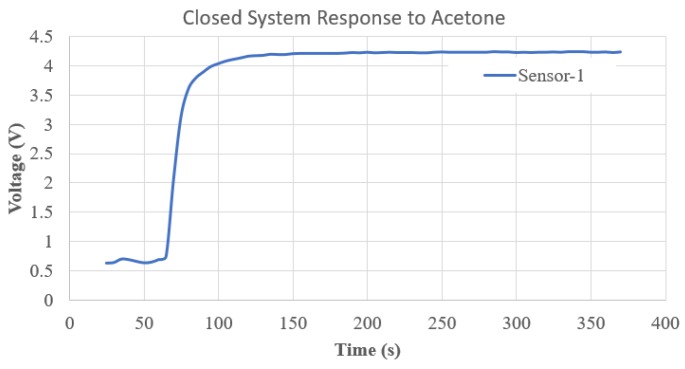
Response of Sensor-1 to acetone over time in an air-tight chamber.

**Figure 2:**
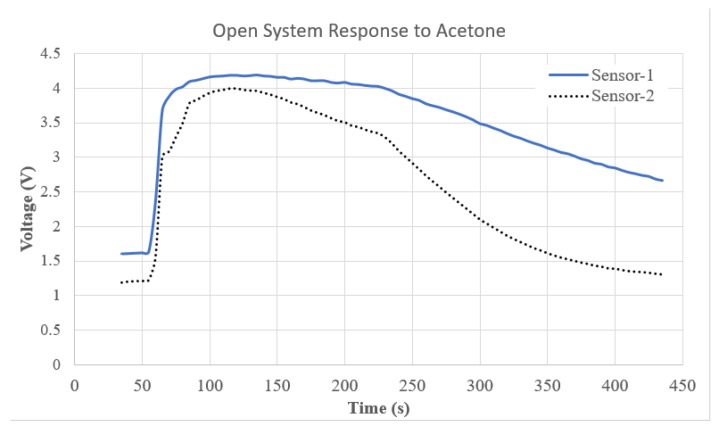
Responses of Sensor-1 and Sensor-2 placed in the third chamber of the system to acetone introduced in the second chamber at 0.5 L/min.

To determine evaporation and diffusion rate for each compound in the chamber, the sensor array was sealed into an air tight chamber after a sample of each acetone or ethanol was introduced separately [28,29]. Figure 1 depicts the response of Sensor-1 to acetone. The result in Figure 1 shows that the sensor began to respond to the acetone 70 s into the experiment. Next, to determine the flow-rate of the system that would yield the maximum quantity of each VOC in the third chamber of the system, where the sensor array would be placed, sensor responses to acetone were measured at a constant air flow (0.5 L/min). Figure 2 shows the response of the sensor array placed in the third chamber when acetone was introduced in the second chamber. This process was repeated for ethanol independently and the results suggest that the peak sensor response for all sensors occurred at the same time as in the acetone test.

**Figure 3:**
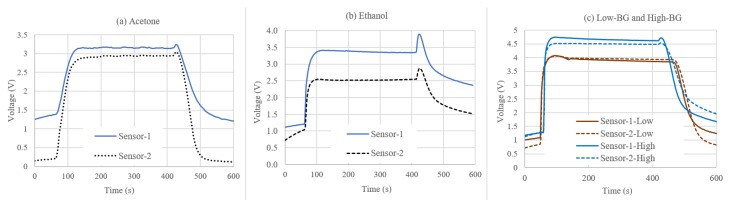
Sample sensor array responses showing results for: (**a**) acetone; (**b**) ethanol; and (**c**) mixed compounds representing low and high blood glucose levels. The spikes in (**b**) seen at the end of steady state and beginning of system clearing cycle were due to the residual ethanol in the VOC chamber, which was transported to sensor array chamber. This also indicated a slower ethanol evaporation.

Based on the above results, a consistent flow control method was developed. The timing and flow control are shown in Table 2. Figure 3 shows the response of the two sensors in the sensor array to 1 ppm of acetone introduced using the process shown in Table 2. While we could not find an exact replica of the tests we conducted with these sensors, comparable experiments are reported in [30,31]. In the published work, the sensors are tested with 1 ppm of acetone and the results are comparable to the sensor responses shown in Figure 3. The sensor responses shown in Figure 3 were divided into five segments, as shown in Table 3, based on the timing of sensor responses to the introduction and evacuation of the VOCs.

## 3. Support Vector Machine

SVM is a machine learning algorithm that has been widely used in published literature for data classification in many areas. For example, SVM has been used to classify tissue samples used for cancer identification [18,19]. SVM was used for this research to evaluate the accuracy of detection of artificial breath samples. The features analyzed by the SVM are straightforward, and a variety of regression techniques or classification techniques could be used. For example, principal component analysis has been used in source classification of indoor air pollutants [32,33]. One could argue that SVM is too sophisticated for this application, but the significance of using SVM to analyze these features is rooted in the portability of this research. The experimental setup used in this research generates a model that can only classify data that are collected using the same setup. A regression-based model would have the same limitations. The benefit of using SVM is that, if this research were continued using human subjects and more sensors, SVM could be trained to achieve the same results that were observed in this experiment, whereas a regression model would have to be restructured entirely to match the new experiment. Thus, the SVM approach for the classification of breath samples makes a greater contribution to the development of a portable biomedical device than a regression approach.

The SVM used in this study was trained to complete a binary classification [34] of artificial breath samples. The presented SVM structure was used in this research because the number of support vectors used in the model required less memory than the data requirements of other machine learning algorithms such as artificial neural networks. SVM requires less memory than the machine learning algorithms discussed in [35]. This makes SVM a better choice for an embedded application with limited storage. The SVM intends to make a distinction between the two diabetes VOC footprints shown in Table 1. The problem in this study required the SVM to generate a two-class solution. The classification function for this problem is defined by Equation (1) where *N* is the number of support vectors used, and $K(x,x_{i})$ is the kernel function chosen. The input parameters *x* and $x_{i}$ are explained when discussing SVM training below. The parameter *b* is the error offset, $a_{i}$ is the data scaling factor, and $y_{i}$ is a dataset that describes the features obtained in testing.

(1) $$f\left(x\right)=\Sigma_{i=0}^{N}a_{i}y_{i}K\left(x,x_{i}\right)+b$$

In training SVM, each entry of datum $x_{i}$ that is entered into Equation (1) is characterized by a class *x*. The kernel function acts as a transformation that generates support vectors that help classify data in Equation (1). In Equation (2), Φ(x) is the kernel transform operation for each class and $\Phi (x_{i})$ is the kernel transform operation for each dataset corresponding to class [34].

(2) $$xx_{i}^{T}\to \Phi \left(x\right)\Phi {\left(x_{i}\right)}^{T}=K\left(x,x_{i}\right)$$

The kernel function maps a nonlinear boundary between all data classes in training while using all support vectors to span the input data across many, or an infinite number of dimensions [34]. There are many models that the kernel function $K(x,x_{i})$ can follow. In this study, a radial basis function (RBF) kernel was chosen. The process used to choose a kernel function for this experiment is based on the work of [36]. The RBF kernel model is described mathematically in Equation (3) where *x* is the class, $x_{i}$ is the dataset corresponding to that class and σ is the variance of the testing data [34,36].

(3) $$K\left(x,x_{i}\right)=exp\left(\frac{-\gamma ||x-x_{i}{\left|\right|}^{2}}{\sigma }\right)$$

The cross-validation method was used to determine the γ and *C* parameters used by the kernel function to scale data and define dimensional space so that the SVM could classify the data most accurately [36]. The cross validation method iteratively attempted to classify a dataset using an SVM that was trained with the remaining data, but, in each iteration, parameters γ and *C* were varied. This generated different support vectors with each iteration and, therefore, a different accuracy in data classification. The goal of the cross validation method was to determine the values of γ and *C* that produced the most accurate results.

## 4. Results and Discussion

Ten experiments were conducted for each VOC separately, and as a combined footprint for low and high BG levels, as indicated in Table 1. Nine tests of each footprint were used to train the SVM, and one test of each class was used for testing. A cross-validation process example is shown Table 4.

The cross-validation process was performed for all data segments defined in Table 3 for results that were generated by testing where acetone, ethanol, and a combination of acetone and ethanol were introduced into the system. The results of the parameters determined by this process can be seen in Table 5, Table 6 and Table 7.

Based on the results of the cross validation process, it was determined that the steady state section of the data yielded the highest accuracy. In addition, little preprocessing was needed to achieve high accuracy classification results for the steady-state samples. The number of features used in training contributed to classification accuracy; however, variations in the transient sections of the experiment were difficult to quantify. For the training and testing of a low and high blood glucose levels, ten sets of results with a combination of acetone and ethanol were used. The two VOCs were introduced sequentially in the first chamber, each with a concentration corresponding to a high or low blood glucose level, as shown in Table 1. The air flow settings shown in Table 2 were used for these tests. The SVM was trained using every permutation of the nine samples from the low BG class and nine samples from the high BG class with the remaining sample from each class used for testing. Data were collected from the experiment every 5 s during the steady state section of the test defined in Table 3. The SVM was able to classify the data collected in this manor with 97.07% accuracy.

In the presented study, we chose diabetes detection as the target application, however, the developed system could be used for other medical and non-medical applications by replacing the sensors used in this study with appropriate sensors and retraining the SVM algorithm. In this study, we used metal oxide semiconductor (MOS) based VOC sensors. These sensors can be replaced by optical and meta-material based sensors, which have also shown to have high sensitivity for applications in medical diagnosis [37,38,39,40]. For example, ref. [40] presented a meta-surfaces based sensing for detecting diabetes, cancer, and blood oxygen level. These and other types of sensors could be used with the presented algorithm for various applications.

The microcontroller program was designed to recognize data from the steady state portion of the experiment in real time tests [41,42]. The same parameters used in the post-processed results were used in the real-time test. For this experiment, new data were collected for testing, and all ten samples of the high BG class and the low BG class were used to train the SVM. New data were collected in the same manner as collected in testing to ensure accuracy. During the steady state portion of the experiment, data were collected from Sensor-1 and Sensor-2 every 5 s to generate a set of 251 total samples each containing two features, one from each sensor. Six total tests were conducted with the microcontroller classifying the data in real time. Three of the tests introduced acetone and ethanol into the system representing a low BG footprint, and the other three tests introduced acetone and ethanol quantities representing a high BG footprint. The microcontroller classified the two features read at 5-s intervals in real time, indicating whether the data were classified as a high BG footprint or a low BG footprint. Of the 753 two-feature readings collected in this manner, 731 of the reading were classified correctly. The other 22 readings were classified incorrectly. These results suggest that this microcontroller experiment was able to classify low BG and high BG VOC footprints in real time with a 97.1% accuracy.

## 5. Conclusions

A microcontroller-based solution that can recognize different VOC footprints consistent with the exhaled breath of patients having high or low blood glucose levels has been presented and discussed. Cross-validation processes were used to determine the SVM parameters and to create a model SVM microcontroller solution that recognizes and classifies data. In real time experiments with artificial breath, the trained model was able to classify low and high blood glucose levels with 97.1% accuracy. These results represent significant progress in developing a noninvasive portable device that could measure blood glucose levels from a patient’s breath.

## Figures and Tables

**Table 1:** VOC concentrations for low and high blood glucose levels [20,21,22].

Compound	Low BG Level	High BG Level
Acetone	1–3 ppm	5–7 ppm
Methyl Nitrate	1 ppm	3 ppm
Ethanol	0–20 ppb	35–50 ppb
Methanol	0 ppb	1 ppb

**Table 2:** Timing and flow control for the chamber.

Time (min)	Action	Air Flow Rate
t = 0–5	Clean System	1.5 L/min
t = 5–6	Introduce Chem.	0 L/min
t = 6–6:45	Blow Chem. to Sensor	0.5 L/min
t = 6:45–12	Steady State Response	0 L/min
t = 12–15	Clear System	1.5 L/min

**Table 3:** Sensor response feature segments.

Feature	Time Segment (s)
Baseline	0–50
Rise	65–85
Steady State	150–400
Fall	450–500
Late Fall	500–600

**Table 4:** Cross-validation process example for acetone.

Acetone	γ = $2^{-3}$	γ = $2^{-2}$	γ = $2^{-1}$	γ = $2^{0}$	γ = $2^{1}$
C = $2^{-3}$	100%	82%	64%	50%	50%
C = $2^{-2}$	82%	82%	63%	50%	50%
C = $2^{-1}$	59%	50%	50%	50%	50%
C = $2^{0}$	68%	59%	50%	50%	50%
C = $2^{1}$	68%	59%	50%	50%	50%
C = $2^{2}$	68%	59%	50%	50%	50%

**Table 5:** Cross validation results for acetone.

Acetone	Baseline	Rise	Steady State	Fall	Late Fall
γ	$2^{-3}$	$2^{-3}$	$2^{-3}$	$2^{-3}$	$2^{-2}$
C	$2^{-3}$	$2^{-1}$	$2^{-3}$	$2^{-1}$	$2^{2}$
Accuracy	100%	73%	100%	77%	78%

**Table 6:** Cross validation results for ethanol.

Acetone	Baseline	Rise	Steady State	Fall	Late Fall
γ	$2^{-3}$	$2^{-3}$	$2^{-3}$	$2^{-1}$	$2^{-3}$
C	$2^{-1}$	$2^{-1}$	$2^{-3}$	$2^{2}$	$2^{-3}$
Accuracy	100%	60%	100%	100%	100%

**Table 7:** Cross validation results for a mixture of acetone and ethanol.

Acetone	Baseline	Rise	Steady State	Fall	Late Fall
γ	$2^{-2}$	$2^{2}$	$2^{-3}$	$2^{2}$	$2^{-3}$
C	$2^{-3}$	$2^{2}$	$2^{-1}$	$2^{-1}$	$2^{1}$
Accuracy	64%	60%	100%	100%	76%

## Abbreviations

The following abbreviations are used in this manuscript:

VOC	Volatile Organic Compound
SVM	Support Vector Machine
ppm	Parts-Per-Million
ppb	Parts-Per-Billion
BG	Blood Glucose
